# Bovine Milk Oligosaccharides with Sialyllactose for Preterm Piglets

**DOI:** 10.3390/nu10101489

**Published:** 2018-10-12

**Authors:** Karina Obelitz-Ryom, Amalie Katrine Rendboe, Duc Ninh Nguyen, Silvia Rudloff, Anne Bladt Brandt, Dennis Sandris Nielsen, Anne Birgitte Heckmann, Maciej Chichlowski, Per Torp Sangild, Thomas Thymann, Stine Brandt Bering

**Affiliations:** 1 Comparative Pediatrics and Nutrition, Faculty of Health and Medical Sciences, University of Copenhagen, 1958 Frederiksberg C, Denmark; karina.ryom@sund.ku.dk (K.O.-R.); dnn@sund.ku.dk (D.N.N.); anne.bladt.brandt@sund.ku.dk (A.B.B.); pts@sund.ku.dk (P.T.S.); thomas.thymann@sund.ku.dk (T.T.); 2 Department of Food Science, Faculty of Science, University of Copenhagen, 1958 Frederiksberg C, Denmark; amkr@ssi.dk (A.K.R.); dn@food.ku.dk (D.S.N.); 3 Institute of Nutritional Science, Justus-Liebig-University Giessen, 35392 Giessen, Germany; silvia.rudloff@ernaehrung.uni-giessen.de; 4 Arla Foods Ingredients, 8260 Viby J, Denmark; anne.birgitte.lau.heckmann@arlafoods.com; 5 Mead Johnson Pediatric Nutrition Institute, Evansville, IL 61142, USA; maciej.chichlowski@rb.com

**Keywords:** sialyllactose, oligosaccharides, preterm neonates, milk, gut, microbiota

## Abstract

Oligosaccharides support gut development and bacterial colonization in term infants, but it is unknown if they benefit preterm infants. Using preterm pigs, we investigated effects of bovine milk supplements enriched with oligosaccharides to improve gut development and colonization. Caesarean-delivered preterm pigs (n = 57) were reared for 19 days. The pigs were fed bovine milk supplemented with an oligosaccharide-enriched whey containing sialyllactose, or a heterogeneous oligosaccharide ingredient. To evaluate the influence of artificial rearing, near-term, vaginally born pigs raised by their sow (n = 12) were compared with artificially reared, caesarean-delivered near-term pigs (n = 14). In preterm pigs, the clinical outcome, gut function, gut microbiota, and systemic immunity were similar among dietary treatments. Natural rearing increased growth rates, gut functions, colon short chain fatty acid concentrations and bacterial diversity, relative to artificial rearing. In conclusion, supplements with bovine milk oligosaccharides were well tolerated, but did not improve gut maturation or clinical outcomes in artificially reared preterm piglets. Immaturity at birth, coupled with artificial rearing, may render the neonate unresponsive to the gut-protective effects of milk oligosaccharides. Whether bovine milk oligosaccharides may affect other endpoints (e.g., brain functions) in conditions of immaturity remains to be investigated.

## 1. Introduction

Preterm birth is the leading cause of newborn death, and more than one million infants worldwide die from preterm birth-related complications annually [1]. For the surviving preterm infants, there is an increased risk for disabilities relative to term infants, and gut immaturity and microbial dysbiosis (higher stool pH, lower production of short-chain fatty acids (SCFA) and increased number of pathogens) may contribute to adverse outcomes [2,3]. Mucosal immunity and appropriate bacterial colonization are key to maintaining gut homeostasis in newborns [4], but little is known about gut colonization in preterm infants and how it may be modulated by diet. The appearance of beneficial bacteria, such as bifidobacteria, may be delayed, relative to colonization in term infants, and with higher densities of potentially pathogenic bacteria [5,6,7]. This disturbed colonization pattern may affect intestinal development in preterm neonates [8].

Breast-fed preterm infants generally have enhanced resistance to infectious diseases and better cognitive function [9,10], and optimal nutrition via breast milk is key to growth, metabolism, and immunity of preterm newborns [11]. Early human milk is a rich source of sialic acid bound to human milk oligosaccharides (HMOs) [12], e.g., sialyllactose (SL), and clinical and preclinical studies have shown that HMOs, including sialyllated oligosaccharides, pass the small intestine undigested and are metabolized in the large intestine [13]. HMOs function as the first prebiotics for infants, but are also suggested to support infant health, growth, and development by acting as antimicrobials, antiadhesives, and modulators of cell responses [14,15,16]. Several beneficial effects of sialylated HMOs related to intestinal function, microbial colonization and immunity in newborns have been documented in vitro and in pre-clinical animal models [17,18,19,20]; for example, the content of disialyllacto-N-tetraose in the milk has been identified as a potential marker of preterm infants at risk of developing necrotizing enterocolitis [21].

The recommendation for feeding of preterm infants is mother’s own milk, or alternatively donor human milk. In cases where neither is available, bovine milk-based formulas are used [22,23,24,25], but addition of HMOs including SL in the formula production is currently not feasible due to lack of bulk amounts [26]. In germ-free mice and newborn piglets, sialylated bovine milk oligosaccharides (BMOs) have shown to change the gut microbiota with altered organ metabolism and improved growth [27]. Similar growth and development was observed in newborn term pigs fed a formula supplemented with 3’SL or 6’SL for 21 days, but with increased brain ganglioside-bound sialic acid in corpus callosum and an altered gut microbiota composition [17]. The effects on growth are most likely related to improved gut metabolism. The 3’SL and 6’SL have been also shown to reduce anxiety-related parameters in juvenile mice [18]. Here, SL supplementation increased the free-to-bound hippocampal sialic acid, reduced bound sialic acid in the prefrontal cortex, and increased mean, axial and radial diffusivity in the corpus callosum in newborn term pigs in a dose-dependent manner, with positive effects at an intermediate dose of 380 mg SL/L milk replacer [19]. While these studies strongly suggest brain developmental effects of dietary SL, the gut-brain mechanisms of these maturational effects remain unknown. Due to a genetic mutation in the human genome, *N*-glycolylneuraminic acid (Neu5Gc) cannot be synthesized [28], and hence the sialylated oligosaccharides in human milk only contains *N*-acetylneuraminic acid (Neu5Ac). In contrast, sialylated oligosaccharides in bovine milk are present both as Neu5Gc (approximately 3–6%, [29,30]) and Neu5Ac. Two recent studies mapping the oligosaccharide composition of porcine milk did not find Neu5Gc in the porcine milk oligosaccharides [31,32], while a third study identified a single milk oligosaccharide linked to Neu5Gc in sow’s colostrum, but not in mature sow’s milk [33]. BMOs, such as galacto-oligosaccharides (GOS), are also considered to have beneficial prebiotic effects in infants and several clinical trials have been conducted with GOS as a supplement to infant formula. The studies showed overall good tolerability but limited clinical effects on growth, gastrointestinal infections, respiratory tract infections and allergic manifestations, but increased stool frequency with a softer stool consistency. Further, changes in microbiota composition has been observed with the increased number of bifidobacteria [34].

The beneficial effects of milk oligosaccharides (MOs) on gut and immune stimulation, growth and brain development as observed in normal term animals and infants may be even more important in sensitive preterm neonates to stimulate gut maturation. However, evidence for beneficial effects of MOs when the intestine is immature at birth is lacking. Recent studies with complex mixtures of HMOs (including sialylated HMOs) only observed trends towards reduced necrotizing enterocolitis in preterm pigs within the first 4–11 days of life, and limited effects on diarrhea, microbial colonization, gut maturation and immune stimulation [35]. We hypothesized that within a slightly longer period of 19 days, MOs may benefit gut and immune maturation and improve gut microbial colonization in preterm neonates. The neonatal pigs share some physiological, anatomical and metabolic characteristics with human infants [36,37]. Furthermore, the composition of milk oligosaccharides in sow’s milk seems to be more comparable to that in human milk than to that in bovine milk [31,32], suggesting that the preterm pig is a suitable model for investigation of effects related to oligosaccharides, even bovine based, in newborns. In the present study (Experiment 1), we tested two industrial bovine milk-based ingredients with improved oligosaccharide content, one enriched with whey-derived oligosaccharides with sialyllactose (SAL) and the other as a pure enzymatically-derived mix of heterogeneous oligosaccharides (HOS). Effects on gut maturation, immunity and microbial colonization within the 19 days were investigated in artificially reared preterm pigs, used as a model for immature infants. The immature immunity of preterm neonates at birth, together with the artificial rearing conditions (e.g., rearing in incubators without their mother, and partial parenteral nutrition) may affect the responsiveness to milk bioactives after birth. Therefore, in an additional experiment (Experiment 2), we evaluated the effects of natural versus artificial rearing by comparing near-term, vaginally born pigs raised by their sow with corresponding artificially reared, caesarean-delivered pigs.

## 2. Materials and Methods

### 2.1. Animal Procedures and Housing

In Experiment 1, 68 pigs derived from three litters were born preterm by cesarean section at day 106 (corresponding to approximately 90% of full gestation), as previously described [38]. Pigs were stratified according to birth weight and allocated to three groups receiving raw bovine milk supplemented with either bovine milk oligosaccharide-enriched whey with sialyllactose (PRE-SAL, n = 22) or an enzymatically-derived mixture of heterogeneous oligosaccharides containing both GOS and N-acetylglucosamine-linked GOS (GlcNAc-GOS) (PRE-HOS, n = 22). For the control group, lactose was added to the milk base (PRE-CON, n = 24). Researchers were blinded to treatment groups throughout the study. Right after birth, the pigs were resuscitated and transferred to oxygenated and temperature-regulated incubators. Within a few hours after delivery, all pigs were equipped with a catheter in the umbilical artery to allow parenteral nutrition (PN) and an orogastric tube for enteral feeding [39]. Within 36 h after birth, all pigs received a total of 25 mL maternal plasma/kg bodyweight divided into 4 intra-arterial infusions to provide passive immunization. The animal procedures have previously been described [38]. Each pig was assessed clinically at least twice daily and assigned a clinical score as described earlier [39]. During the first 11 day, oral antibiotics were given prophylactic (ampicillin, Norobrittin Vet. 30 mg/kg three times daily and gentamycin, Gentocin Vet., 2.5 mg/kg twice daily, both ScanVet, Fredensborg, Denmark). On day 2, all pigs received a subcutaneous injection of iron (Uniferon, 30 mg per pig, Pharmacosmos, Holbæk, Denmark). A total of 99% of the pigs suffered from various degrees of diarrhea during the study period. Mild cases of diarrhea were supported with an electrolyte supplement (Revolyt nutrition, Gunnar Kjems, København, Denmark), while more severe cases of diarrhea were treated with oral antibiotics (ampicillin and gentamycin) for three consecutive days. In cases with marked clinical affection, additional treatment with systemic antibiotics (ampicillin, Pentrexyl, 30 mg/kg, Bristol-Myers Squibb, Solna, Sweden and gentamicin, Hexamycin, 2.5 mg/kg, Sandoz, Copenhagen, Denmark), as well as an addition of metronidazole (Flagyl, 10 mg/kg, sanofi-aventis, Hørsholm, Denmark) to the oral treatment regime, was initiated. Eleven pigs were euthanized during the study period due to clinical complications unrelated to the experimental diets; hence, these data were not included in the analysis. The final group sizes were PRE-SAL (n = 19), PRE-HOS (n = 18) and PRE-CON (n = 20).

In Experiment 2, one litter of pigs was born on day 112 (e.g., near the anticipated term date of 116–117) by spontaneous vaginal delivery and these pigs were allowed to suckle the sow under natural rearing conditions in the experimental facility (TERM-NAT, n = 12). Following birth, the near-term pigs were mobile, thermoregulatory stabile and had a normal ability to suckle their sow (in contrast to preterm pigs delivered at 90% gestation). These pigs were compared with pigs delivered from a sow by cesarean section close to term (day 115). The latter pigs were reared under the same conditions as the preterm pigs in Experiment 1 and fed the same diet (TERM-CON, n = 14).

### 2.2. Diets

All experimentally reared pigs (both preterm and near-term) received an enteral base milk diet supplemented with HOS, SAL or only the control raw bovine milk (CON). All pigs started minimal enteral nutrition (MEN) at 16 mL/kg/day on the day of birth increasing gradually to 180 mL/kg/day on day 16. The advancing volumes of enteral nutrition were based on experience from a similar type of studies in our lab [39] and reflected the upper limit of what was tolerable in the immature intestine of these preterm pigs. During the first seven days, the pigs were supplemented with PN, 48–144 mL/kg/day (Kabiven modified with Vamin, Soluvit, Vitalipid and Peditrace, all Fresenius Kabi, Uppsala, Sweden) to meet specific nutrient needs of preterm pigs [38]. During the first five days, enteral feeding was administered exclusively through the fitted orogastric tubes. From day five onwards, the pigs were introduced to the milk in drinking troughs and were assisted with the drinking until they mastered the task on their own. The milk was supplied via the orogastric tube until full enteral boluses could be consumed voluntarily by each pig. The naturally reared pigs (TERM-NAT) received the sow’s milk only.

The enteral diet consisted of intact unpasteurized Jersey cow’s milk (protein 38.2 ± 0.35 g/L, lipid 31.65 ± 1.83 g/L and carbohydrate 44.4 ± 0.44 g/L). After collection from the farm, the milk was stored in aliquots at −20 °C. The HOS blend contained a mixture of GOS and GlcNAc-GOS produced by trans-galactosylation of bovine milk-based lactose and GlcNAc added as a secondary substrate (6.7 g/L, Arla Foods Ingredients, Viby, Denmark). The HOS diet was prepared by mixing the HOS blend as well as lactose (6 g/L, Variolac^®^ 960, Arla Foods Ingredients, Viby, Denmark) with the milk, to a final dose of 5 g oligosaccharides per liter of milk. The SAL diet was prepared by adding the SAL blend (8.5 g/L Lacprodan SAL-10^®^, Arla Foods Ingredients, Viby, Denmark) to the milk, to a final dose of 380 mg SL per liter of milk. The ingredient contained 4.5% SL with a 6:1 composition of 3’SL and 6’SL respectively. Additionally, other acidic and neutral bovine milk oligosaccharides from whey were also present in the product (not quantified). To secure isocaloric diets, 6 g lactose (Variolac^®^ 960, Arla Foods Ingredients, Viby, Denmark) was added per liter milk of the raw milk in the CON diet. Milk was prepared on a daily basis and stored at 4 °C until use. Aliquots were heated to a maximum temperature of 40 °C prior to feeding.

Verification of the total dosage of SL given in the milk diets, including 3’SL and 6’SL, were determined using High Performance Anion Exchange Chromatography—Pulsed Amperometric Detection (HPAEC-PAD). The total levels were 0.433 ± 0.005 mg/mL and 0.120 ± 0.002 mg/mL in the SAL and CON diets, respectively, with a ratio of 3’SL and 6’SL of 5:1.

### 2.3. Body Composition and Tissue Collection

On day 19, the pigs were anesthetized with an intramuscular injection of a mixture with zolazepam/tiletamin (Zoletil 50, Virbac, Kolding, Denmark), xylazine (Xysol, ScanVet, Fredensborg, Denmark), ketamine (Ketaminol, MSD Animal Health, Copenhagen, Denmark) and butorphanol (Torbugesic, ScanVet, Fredensborg, Denmark). For each pig, the body composition was determined by Dual Energy X-ray absorptiometry (DEXA, Lunar Prodigy scanner, GE Healthcare, Little Chalfont, UK). All scannings were performed in ventral recumbency. Fat and lean mass percentages plus bone mineral density and bone mineral content were estimated using software provided with the DEXA scanner. Before euthanasia, blood samples were drawn intracardially. All pigs were euthanized with an intracardiac injection of sodium pentobarbital (Euthanimal, Scanvet, Fredensborg, Denmark). Cerebrospinal fluid (CSF) was collected by suboccipital puncture, collected in cryovials and frozen in liquid nitrogen. Organ weights were measured and intestinal samples (proximal, middle and distal intestine and colon) were dissected and either frozen in liquid nitrogen or immersed in Clarke’s solution (absolute alcohol and glacial acetic acid) for fixation before histological preparation. Femurs from the right hind leg of all the preterm pigs were dissected from the tissue and immersed in ethanol before aseptic extraction of the bone marrow caput femur for culture. The bone marrow was homogenized in 1 mL sterile saline using a Stomacher (Seward Ltd., Worthing, UK) and the homogenate was plated (1:1, 1:10 and 1:100 v/v dilutions in sterile saline) on tryptic soy agar with 5% sheep blood, and incubated for 24 h at 37 °C. For positive cultures, bacterial isolates would be identified to the species level by matrix-assisted laser desorption/ionization time-of-flight (Maldi-TOF) mass spectrometry (Vitek MS RUO, bioMerieux; Craponne, France). All experimental procedures conducted during the animal experiments were approved by the Danish Animal Experiments Inspectorate (license number 2014-15-0201-00418), which is in accordance with the guidelines from Directive 2010/63/EU of the European Parliament and the Animal Research: Reporting of In Vivo Experiments (ARRIVE) guidelines [40].

### 2.4. Gut Structure

Villus height and crypt depth were measured by staining with Alcian Blue-Periodic Acid Shiff (AB-PAS) on Clarke’s fixated samples from the distal part of the small intestine, and the villus-crypt ratio and colonic goblet cell density was calculated [41]. Pictures were obtained at 200× magnification by light microscope (Olympus BX45TF, Tokyo, Japan) and a camera with belonging software, cell^A (version 3.4, Olympus, Tokyo, Japan). The goblet cell density was calculated as the area fraction covered by goblet cells relative to the total area covered by tunica mucosa (STEPanizer stereology tool, version 1.0, STEPanizer, Bern, Germany).

### 2.5. Gut Function

The digestive capacity for lactose, a surrogate marker of mucosal function, was assessed on days 10 and 19 by an oral administration of a 5% lactose solution (15 mL/kg). The increment in blood galactose was measured after 40 min from a jugular blood sample [42]. The intestinal permeability was assessed after an oral administration of a 5% lactulose and 5% mannitol solution 3 h before euthanasia. At the time of euthanasia, urine was collected by intra-abdominal cystocentesis. The urine was stored at −20 °C until spectrophotometrical analysis [42]. Activities of the brush border disaccharidases, lactase, maltase, and sucrase, as well as the peptidases, aminopeptidase A (ApA), aminopeptidase N (ApN), and dipeptidyl peptidase IV (DPPIV), were measured by spectrophotometry of tissue homogenates originating from the proximal, middle and distal parts of the small intestine [43]. Enzyme activities were expressed as units per gram of wet tissue.

### 2.6. Microbiome and Microbial Metabolites

Total DNA was extracted from samples of colon content collected at euthanasia using the Powersoil DNA isolation kit (MoBio Laboratories, CA, USA) according to the manufacturers description including an initial bead-beating step using a FastPrep-24™ 5G (MP Biomedicals, CA, USA). The V3V4 region of the 16S rRNA gene was amplified, sequence adapters and indices were incorporated, the final amplicon constructs were purified and the library was pooled as previously lined out [44]. The library was subsequently sequenced by tag-encoded 16S rRNA gene MiSeq-based (Illumina, CA, USA) high-throughput sequencing as described in detail previously [44]. The raw dataset was analyzed as previously described [44] to obtain operational taxonomic unit (OTU) tables, alpha diversity plots (expressed as observed species) and UniFrac Distance metrics principal coordinate analysis (PCoA) plots for beta diversity. Analysis of similarities (ANOSIM) was used to evaluate group differences using weighted and unweighted UniFrac analysis. The G test of independence (q_test) was used for qualitative assessments of the OTUs and analysis of variance (ANOVA) for the quantitative associations (relative abundance).

Levels of microbial metabolites and short-chain fatty acids, including acetic acid, butyric acid, propionic acid, valeric acid, propanediol and methyl butanoic acid, were measured in colon content by gas chromatography–mass spectroscopy and data were presented as μmol/g of wet matter [35]. Lactic acid levels were measured by an injection of 10 µL of supernatant into an Agilent LC/MS 1100 system, equipped with a Repromer H column (250 × 8 mm, 9 µm with matching guard; Maisch, Ammerbuch, Germany). The mobile phase consisted of isocratic sulfuric acid (5 mmol/L) heated to 50 °C for columns and detectors with a flow rate of 0.5 mL/min [45]. Quantitation was done by refractive index detection (RID; Agilent 1260 Infinity II) using lactic acid (Sigma-Aldrich, Taufkirchen, Germany) as an external standard.

### 2.7. Hematology, Biochemistry and Systemic Immunity

Heparinized blood, obtained from either cord blood at the cesarean section or by jugular samples at days 10 and 19, was used for blood cell counting by an automatic cell counter (Advia 2120i Hematology System, Siemens Healthcare GmbH, Erlangen, Germany). Blood and CSF biochemistry was analyzed on serum from blood obtained before euthanasia and CSF obtained after euthanasia (ADVIA 1800 Chemistry System, Siemens Healthcare GmbH, Erlangen, Germany). Immune cell phagocytosis in preterm and TERM-CON pigs was performed using pHrodo green *Staphylococcus aureus* (509/533 nm) BioParticles Phagocytosis Kits (Life Technologies, Taastrup, Denmark) for flow cytometry [46]. In brief, 100 µL whole blood was challenged with pHrodo Green-conjugated *S. aureus* (ratio of particle-to-phagocyte approximately 10:1) at 37 °C, 5% CO_2_ for 30 min. Red blood cells were then lyzed, and the remaining leukocytes were washed with PBS prior to analysis by FACS Canto II flow cytometer (BD biosciences, San Jose, CA, USA). Samples without *S. aureus* challenge served as controls. The neutrophil population was gated based on forward scatter (FSC) and side scatter (SSC) dot-plots. Neutrophils with pHrodo+ were identified as neutrophils exerting phagocytic function. Fluorescence intensity of pHrodo+ population indicated the number of bacteria being phagocytized.

### 2.8. Statistics

Data were analyzed with the software package R (version 3.3.2, R Foundation for Statistical Computing, Vienna, Austria), unless otherwise stated, and group comparisons were performed. Continuous data were analyzed with mixed models adjusting for sex and birth weight as fixed variables and litter as a random variable. Repeated measurements were analyzed by the lmer function for mixed modeling as repeated measures. All statistical models were validated by assessment of residuals and fitted values for normality as well as variance homogeneity. The microbiome data were analyzed as previously described, including false discovery rate (FDR) correction [44]. Genera accounting for >1% of the total relative abundance were presented graphically. *p* < 0.05 was chosen as the critical level of significance. Data were presented as raw arithmetic means and SD, unless otherwise stated. For data interpretation, the preterm intervention groups, PRE-SAL and PRE-HOS, were compared with the preterm control group (PRE-CON). Rearing conditions were compared by comparing TERM-CON with TERM-NAT.

## 3. Results

### 3.1. Experiment 1

#### 3.1.1. Clinical Condition, Body Growth and Composition

**Figure 1:**
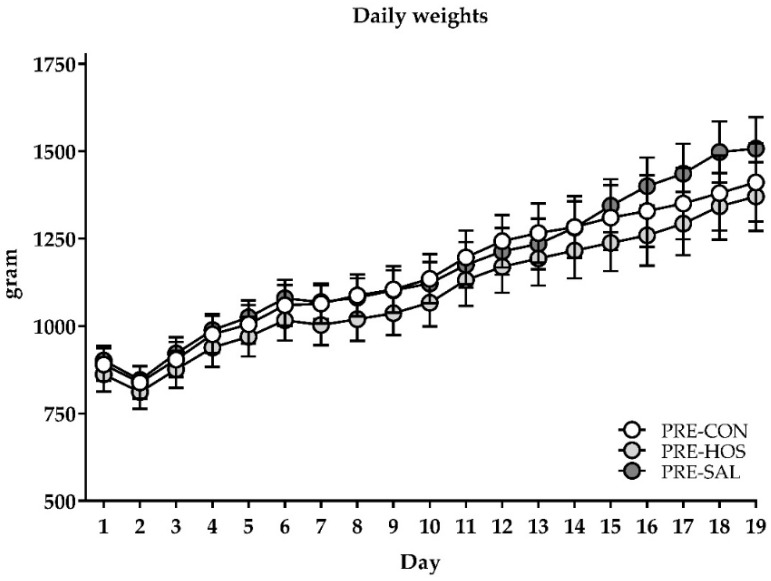
Growth curves in preterm pigs (Experiment 1). Daily weights of preterm pigs with or without oligosaccharide supplementations. No differences were observed between PRE-CON (white) and the supplemented groups, PRE-HOS (light grey) and PRE-SAL (dark grey). Values are presented as mean ± standard error of the mean (SEM).

Birth weight and body weight gain were similar across PRE-CON, PRE-HOS and PRE-SAL, with an average birth weight of 896 ± 196 g, and an average body weight gain of ~24 g/kg/day across the groups (Figure 1). Further, they had similar bone mass density and bone mineral content on day 19 as well as equal fat and lean mass percentages (Figure S1, Supplementary Materials).

There were episodes of diarrhea among PRE-CON, PRE-HOS and PRE-SAL with a mean of 8.7 days with diarrhea for all groups. Particularly, one of the three litters of preterm pigs showed marked diarrhea and was severely clinically affected (on average 13.3 days with diarrhea relative to 6.7 and 6.8 days for the other two litters). This litter was treated systemically with additional antibiotics and received increased volumes and 5-day extension of the PN support to ensure survival of the pigs. Bone marrow homogenates collected on day 19 from all preterm animals were sterile, indicating that no pigs had blood stream infection at this time point. Relative to PRE-CON, the PRE-SAL had lower relative weights of the adrenal glands (*p* < 0.05), whereas the relative weights of other organs did not differ among the preterm groups (Table S1, Supplementary Materials).

#### 3.1.2. Gut Structure and Function

**Figure 2:**
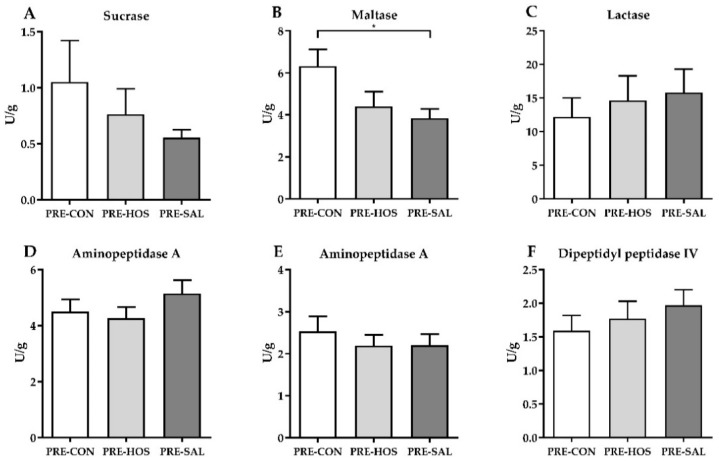
Small intestinal brush border enzyme activities in preterm pigs (Experiment 1). (**A**) Sucrase, (**B**) maltase, (**C**) lactase, (**D**) aminopeptidase N, (**E**) aminopeptidase A and (**F**) dipeptidyl peptidase IV. All were measured in the middle part of the small intestine. Values are presented as mean ± SEM. The * indicate statistical significant difference between groups of *p* < 0.05.

No differences were observed among the preterm groups for villus and crypt lengths as well as villus–crypt ratios, and the goblet cell density was similar between the three groups (Figure S2, Supplementary Materials). The increment in plasma galactose after an oral lactose bolus given on days 10 and 19 did not differ between the three preterm groups (61 ± 60 µmol/L and 101 ± 164 µmol/L, respectively, across groups). Similarly, the intestinal permeability measured by the lactulose-to-mannitol ratio on day 19 was equal among the groups (0.50 ± 0.56 across groups), as well as the activity of the six different brush border enzymes in the proximal and distal part of the small intestine. In the middle part of the small intestine, PRE-SAL had decreased maltase activity relative to PRE-CON (*p* < 0.05, Figure 2).

#### 3.1.3. Gut Microbiota and Microbial Metabolites

**Figure 3:**
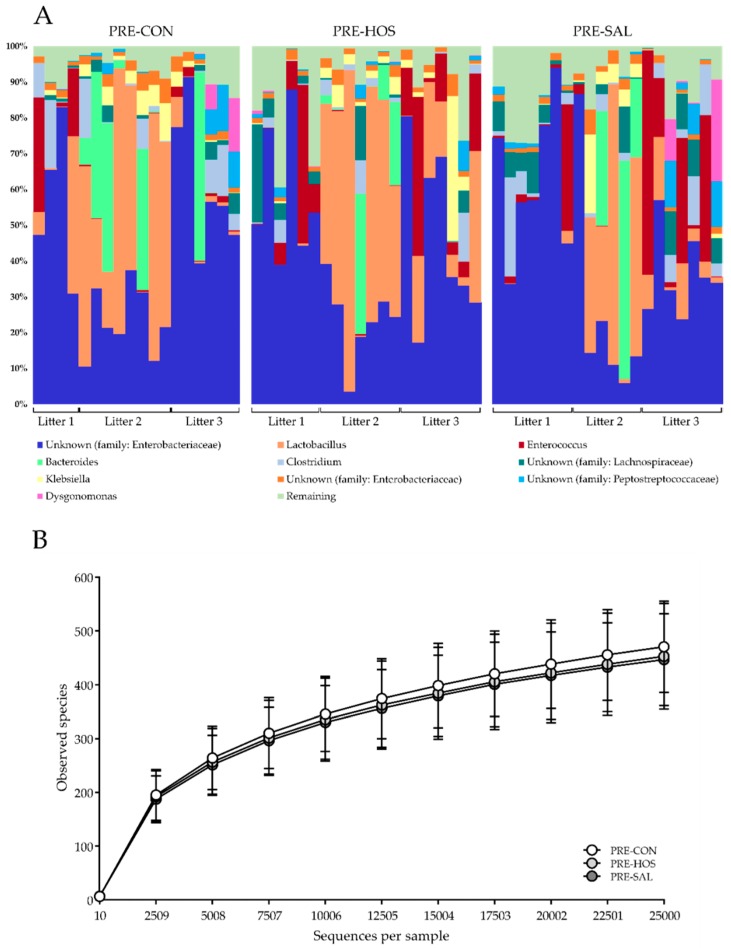
Microbiology in preterm pigs (Experiment 1). (**A**) Relative microbial abundance in colon for each preterm pig as determined by 16S rRNA gene amplicon sequencing. (**B**) Microbial diversity in colon of preterm pigs shown by the number of observed species. No differences were observed between PRE-CON (white) and the supplemented groups, PRE-HOS (light grey) and PRE-SAL (dark grey), respectively. Values are presented as mean ± SD.

For the gut microbiota analyses by 16S rRNA gene amplicon sequencing, five samples out of a total of 88 samples were excluded due to low sample reads (<25,000 reads per sample). The colonic microbial diversity at day 19, as indicated by the number of bacterial OTUs, was not influenced by the different feeding protocols in the preterm pigs. The majority of sample reads (>99%) were distributed within five different phyla, with Proteobacteria (mainly represented by family Enterobacteriaceae) and Firmicutes (with *Lactobacillus* spp. and *Enterococcus* spp. among the dominating species, together with members of the family Lachnospiraceae) dominating the gut microbiota (Figure 3A). No differences among the preterm groups were observed for the number of observed species (Figure 3B). Neither was the colonic microbiota composition influenced by the different feeding protocols, as determined by both weighted as well as unweighted UniFrac distance dissimilarity metrics (results not shown).

**Figure 4:**
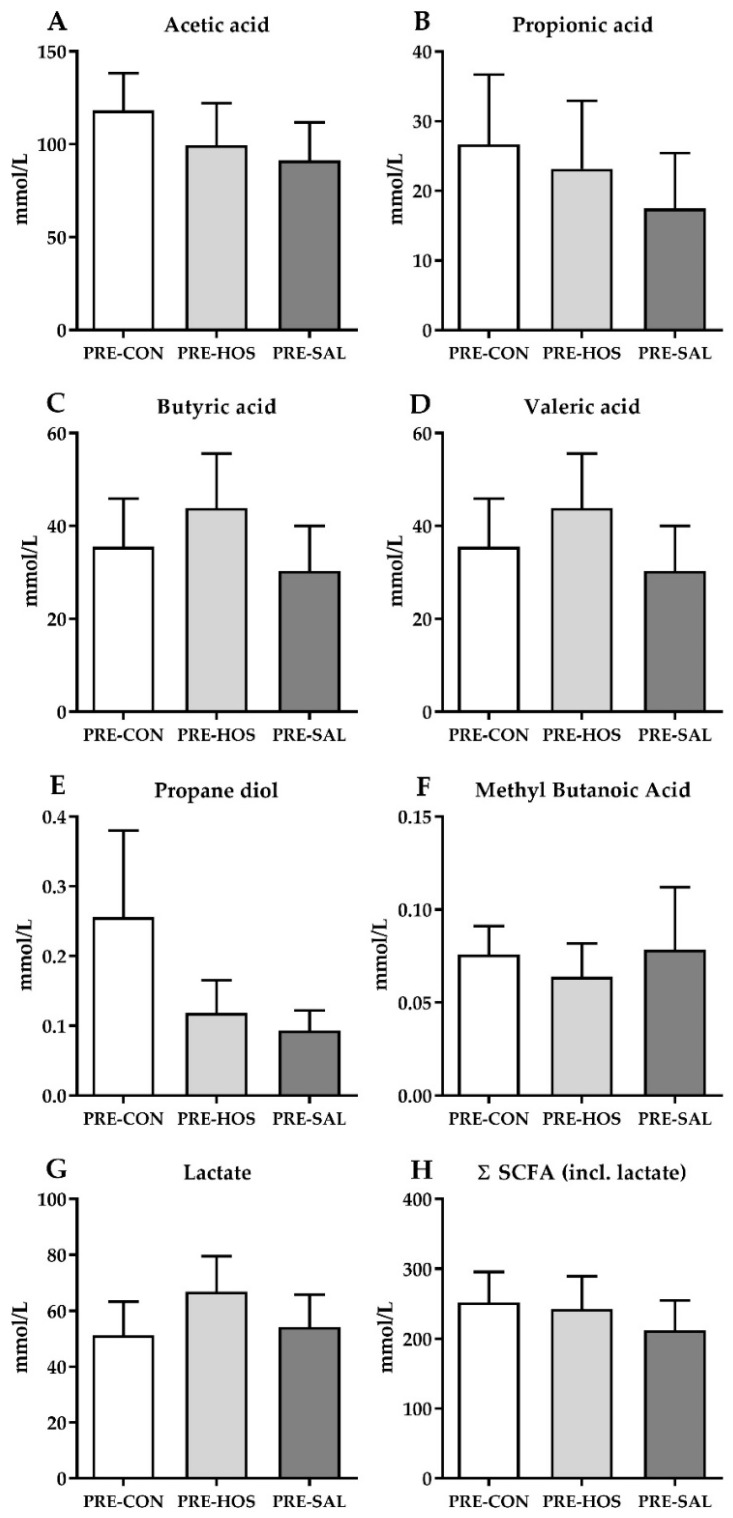
Microbial metabolite concentrations in colon content in preterm pigs (Experiment 1). (**A**) Acetic acid, (**B**) propionic acid, (**C**) butyric acid, (**D**) valeric acid, (**E**) propanediol, (**F**) methyl butanoic acid, (**G**) lactate (hydroxy propanoic acid) and (**H**) sum of all microbial metabolites including lactate (Σ(SCFA)). Values are presented as mean ± SEM.

The levels of the microbial metabolites, acetic acid, butyric acid, propionic acid, valeric acid, propanediol, methyl butanoic acid and lactate were all similar between PRE-CON, PRE-HOS and PRE-SAL (Figure 4).

#### 3.1.4. Hematology, Biochemistry and Systemic Immunity

Blood cell counts on days 1, 10 and 19 are listed in Table S2 (Supplementary Materials). Apart from PRE-SAL, which had higher platelet counts at birth relative to PRE-CON (*p* < 0.05), no differences were observed between the three preterm groups. Serum biochemistry on day 19 showed increased values of alkaline phosphatase and glucose along with lower values of aspartate aminotransferase in PRE-HOS, relative to those in PRE-CON (all *p* < 0.05, Table 1). No differences in serum biochemistry parameters were observed between PRE-CON and PRE-SAL, whereas the CSF biochemistry showed higher values of glucose for PRE-HOS compared with those for PRE-CON (*p* < 0.05).

**Figure 5:**
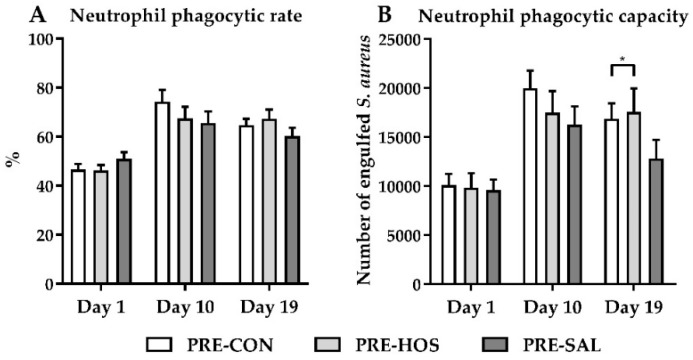
Phagocytic activity in preterm pigs (Experiment 1). (**A**) Proportion of neutrophils exerting phagocytosis against *Staphylococcus aureus* and (**B**) total number of engulfed *S. aureus* by neutrophils. Values are presented as mean ± SEM. The * indicate statistical significant difference between groups of *p* < 0.05.

PRE-CON, PRE-HOS and PRE-SAL had similar proportions of neutrophils with phagocytic capacity of *S. aureus* as measured in cord blood at day 1, and the phagocytic capacity increased after birth in a similar manner between the groups (Figure 5A). At day 19, the number of engulfed *S. aureus* in PRE-HOS was higher relative to PRE-CON pigs (*p* < 0.05, Figure 5B).

### 3.2. Experiment 2

#### 3.2.1. Clinical Condition, Body Growth and Composition

The naturally reared TERM-NAT were weighted on day 3 after birth, with a mean body weight of 1298 ± 311 g, which was higher than the artificially reared TERM-CON on day 3 (1133 ± 166 g, *p* < 0.001), and had a higher average weight gain (~64 g/kg/day) relative to TERM-CON (~22 g/kg/day, *p* < 0.001). The TERM-CON had an average of 6.4 days with diarrhea, whereas TERM-NAT had no clinical complications during the study period. The bone mass density was lower in TERM-CON compared to TERM-NAT (0.201 ± 0.016 g/cm^2^ vs. 0.244 ± 0.042 g/cm^2^, *p* < 0.05). In contrast, the relative bone mineral content and lean mass percentage were higher in TERM-CON (17.7 ± 1.48 g/kg and 94.1 ± 7.37%) relative to TERM-NAT (15.0 ± 1.04 g/kg and 88.9 ± 6.06%, *p* < 0.001 and 0.01, respectively). The fat percentage was lower in TERM-CON (2.71 ± 1.17%) relative to TERM-NAT (8.56 ± 5.13%, *p* < 0.01). The relative weights of the proximal small intestine, colon, adrenals and brain were higher in TERM-CON relative to TERM-NAT pigs, while the weights of the middle part of the small intestine, spleen and lungs were lower (Table S3, Supplementary Materials).

#### 3.2.2. Gut Structure and Function

TERM-CON had shorter crypts and villi relative to TERM-NAT (*p* < 0.01), as well as a lower villus–crypt ratio (6.88 ± 2.76 vs. 16.0 ± 4.69, *p* < 0.001). The goblet cell density was increased (11.8 ± 4.56%) relative to TERM-NAT (6.81 ± 3.27%, *p* < 0.01). The intestinal permeability was equal among the two near-term groups (lactulose mannitol ratio of 0.55 ± 1.05 in both groups), whereas TERM-CON had decreased enzyme capacities for all measured enzymes relative to TERM-NAT (71.2–97.2% decrease, all *p* < 0.001, Table S4, Supplementary Materials).

#### 3.2.3. Gut Microbiota and Microbial Metabolites

**Figure 6:**
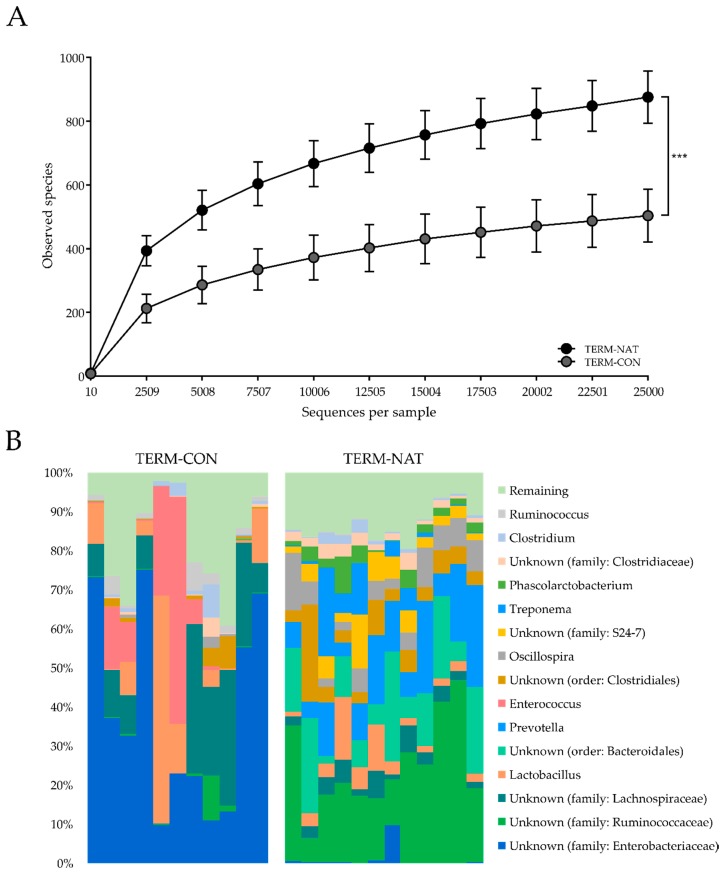
Microbiology in near-term pigs (Experiment 2). (**A**) Microbial diversity in colon of near-term pigs shown by the number of observed species. (**B**) Relative microbial abundance in colon for each near-term pig as determined by 16S rRNA gene amplicon sequencing. Values are presented as mean ± SD. The *** indicate statistical significant difference between groups of *p* < 0.001.

The number of observed species was higher in TERM-NAT (*p* = 0.001, Figure 6A) compared to TERM-CON, and they were dominated by Firmicutes (mainly members of the Ruminococcaceae family), Bacteroidetes (mainly represented by the Bacteroides and Prevotella genera) and the phylum Spirochaetes (<5%) (Figure 6B), whereas TERM-CON pigs were distributed similarly to the preterm pigs in Experiment 1, raised in the same artificial environment. The colonic microbiota composition of TERM-NAT was different from that of TERM-CON, as determined by both weighted as well as unweighted UniFrac distance dissimilarity metrics (*p* < 0.001, results not shown). Lactate levels were higher in TERM-CON (849% higher, *p* < 0.01), but levels of the other microbial metabolites measured, except for propanediol, were lower relative to TERM-NAT pigs (52–98% lower, *p* < 0.01, Table S4, Supplementary Materials). The total amount of microbial metabolites measured tended to be lower in TERM-CON relative to in TERM-NAT (*p* = 0.053, Table S4, Supplementary Materials).

#### 3.2.4. Hematology, Biochemistry and Systemic Immunity

The capacity of neutrophils to engulf *S. aureus* was 2.6-fold higher than the preterm pigs in Experiment 1, indicating that the systemic immunity in TERM-CON pigs at birth was more mature. At day 10, TERM-CON showed lower counts of platelets and white blood cells (WBC), including neutrophils, lymphocytes and monocytes relative to TERM-NAT, but had higher counts of red blood cells and basophils as well as an increased level of haemoglobin. At day 19, TERM-CON further showed an increased hematocrit relative to TERM-NAT (*p* < 0.01, Table S5, Supplementary Materials). Many serum biochemistry markers (e.g., albumin, creatinine and cholesterol) differed between the TERM-CON and TERM-NAT, and the CSF biochemistry showed lower values of both albumin and total protein in TERM-CON relative to TERM-NAT (*p* < 0.01, Table S6, Supplementary Materials).

## 4. Discussion

Enteral feeding is key to proper organ development, particularly in preterm neonates. Due to the presumed beneficial effects of MOs, the addition of bovine milk-based oligosaccharide-enriched whey to the milk diet within the first weeks of life could potentially be beneficial for preterm newborns that do not receive human milk, by stimulating immune and microbial parameters. Optimally, when human milk is not available, oligosaccharide structures, similar to the ones found in human milk, should be added to infant formula to simulate the human milk patterns. Ddue to the high species variation and low production capacity this has so far been difficult. While sialic acid in human milk is mainly bound to oligosaccharides, cow’s milk-based infant formula has the majority of sialic acid bound to glycoproteins [26]. Limited research has investigated effects of oral administration of sialic acid or SL in preterm neonates. We hypothesized that a bovine milk-based ingredient enriched in oligosaccharides, including SL in levels similar to that of mature human milk, would benefit several gut and immune maturation parameters. Similarly, an oligosaccharide ingredient produced enzymatically from bovine lactose and GlcNAc represents a slightly more complex mixture of heterogeneous oligosaccharides as compared to BMOs.

In this study, we showed that pigs fed SAL and HOS tolerated the experimental diets with no additional adverse effects relative to PRE-CON. The tolerability is in agreement with the literature within this field [17,47,48,49,50]. PRE-SAL pigs, which received oligosaccharides with SL in levels equal to the natural content of 330–880 mg/L in human breast milk [51,52,53], in general, did not differ in parameters related to growth, organ weights, microbiota, functional gut parameters and immunity compared to PRE-CON. This is in line with earlier studies. In rats, a subchronic toxicity study with oral administration of a synthetically produced sialic acid showed small negative changes in body weight, but overall, no signs of adverse effects due to administration of sialic acid were observed [47].

Similar growth curves were observed for PRE-SAL, PRE-HOS and PRE-CON, and analysis of the body composition after DEXA scans did not reveal any differences among the three preterm groups. Values on day 19, for both the bone mineral content, lean mass and fat percentage, are comparable with results from 26-day-old preterm pigs [39]. The difference in platelet counts between PRE-CON and PRE-SAL was only seen on day 1 and was a coincidental result from the stratification and not diet-related. All three preterm groups had similar gut structural values for both the crypt and villus measures in the distal small intestine as well as the goblet cell density in the colon. Among the brush border enzymes, only PRE-SAL had lower activity of maltase relative to PRE-CON in the middle part of the small intestine. Since no other gastrointestinal changes were observed, we considered this an occasional finding. Brush border enzyme activities in the present study were similar to the levels measured in a previous study with preterm pigs reared for 26 days [54]. All groups showed similar functional gut properties with regard to absorptive capacity and intestinal permeability with no additional effects of the bovine milk oligosaccharides.

The main effects of oligosaccharides in the neonatal gut have been related to modulation of the microbiota as well as their fermentation products by either direct or indirect effects [55]. The microbiota analysis of colon content in the pigs on day 19 revealed a similar overall pattern across all three preterm groups. Collectively, the relative abundance as well as similar alpha and beta diversity suggested that addition of oligosaccharide-enriched whey did not have additional effects on the bacterial gut microbiota in this study. This is in contrast to an earlier study where changes in the colonic microbiota were observed [17]. Further, no differences in species richness or overall patterns of OTUs were observed between PRE-CON, PRE-SAL or PRE-HOS. Due to the occurrence of diarrhea in many of the pigs, they were treated with antibiotics. Administration of antibiotics in early life to preterm infants has profound impact on the microbiota, including decreased diversity and lowered colonization beneficial bacteria, such as *Bifidobacterium*, while potentially undesirable taxa, such as *Enterococcus*, increase [56]. Thus, the antibiotics treatment may possibly have surpassed any potential positive influence of the supplemented oligosaccharides on the microbiota. Further, the levels of microbial metabolites were similar among all three preterm groups, suggesting an unaltered fermentation pattern after oligosaccharide supplementation. The production of SCFAs depends on both the species and the number of bacteria present in the colon [57], as well as substrate availability. The antibiotics treatment administered during the artificial rearing and the immature gut epithelium is possibly determining the microbiota and thereby the amount of SCFAs [58,59,60]. The relatively short exposure time (19 days) to the MOs may be too short to produce detectable differences between the preterm groups, although it represents most of the milk feeding period of pigs. The SL presented as Neu5Gc in bovine milk is only present in limited amounts in porcine milk [31,32,33], but is found in pork meat [26,61]. Even though Neu5Gc content is minor in porcine milk and not present in human milk, it may serve similar activity related to the external gut environment, such as the microbiota, which remains more universal across species.

Relative to the preterm pigs, the TERM-CON had a higher proportion of neutrophils with phagocytotic capacity both at birth and day 19. This observation confirmed that the systemic innate immunity of the preterm pigs was immature relative to their near-term born counterparts [4], making them more susceptible to infections.

As a consequence of preterm birth, the lacking organ maturity and abnormal microbial colonization puts the infant at higher risk of adverse outcomes [2,3]. TERM-CON was born with a higher birth weight relative to their preterm counterparts, and the higher body weight remained consistent throughout the study period, although similar growth rates were observed for the preterm and near-term pigs. Both the preterm pigs and TERM-CON suffered from diarrhea during the study period, but surprisingly, the extent and clinical consequences were more profound in the TERM-CON group. Compared with other studies of term pigs born and reared in our lab in an identical manner [39], the clinical complications and low growth rates observed in the present study may reflect that the TERM-CON litter was particularly compromised. The TERM-CON further had lower fat percentages, reflecting emaciation, but higher bone mineral densities compared with the preterm pigs. In comparison, the near-term pigs born by vaginal birth, and kept and nourished by the sow (TERM-NAT) had markedly higher weight gain and no diarrhea. DEXA scans revealed both lowered bone mineral densities as well as a decreased fat percentage for TERM-CON relative to TERM-NAT. The differences in gut functions further substantiate the general difference between TERM-CON and TERM-NAT, as TERM-CON showed both decreased villus length and crypt depths in the small intestine as well as a marked higher goblet cell density in colon. This was also reflected on the functional side, where TERM-CON had decreased activities of all six brush border enzymes compared with TERM-NAT. As expected, many environmental factors (delivery mode, rearing conditions, medication and nutrition) lead to different microbiota composition as well as changes in the concentration of microbial metabolites. TERM-NAT showed a higher diversity and increased levels of microbial metabolites, other than lactate, compared to TERM-CON. This reflects a mature and fully functional anaerobe flora with a lower degree of facultative anaerobes and less *Lactobacillus* spp.

These results implied that environmental factors have a major influence on early life development, and although it is not possible to strictly differentiate between litter effects and environmental effects, the profound difference between the two rearing conditions was demonstrated. The TERM-NAT group showed the physiological potential of near-term pigs when rearing conditions were optimal. In contrast, the TERM-CON group delineated the neonatal responses of artificial rearing conditions identical to the three preterm groups, which may pose a stronger mark on development of the neonate than optimized nutrition can influence.

## 5. Conclusions

In conclusion, supplementation of bovine milk-based oligosaccharide ingredients did not improve gut and immune endpoints in preterm pigs during the milk feeding period. The artificial rearing conditions of preterm pigs in incubators led to clinical complications, and the supplements did not reduce or increase the clinical symptoms. The changes in growth, body composition and gut function were more affected by the mode of delivery and artificial rearing rather than by gestational age at birth. Potential beneficial effects of oligosaccharide-rich bovine milk supplements on gut and immune functions may be less effective following preterm birth and artificial rearing, compared with more natural conditions. The effects on other endpoints, such as brain functions, in conditions of immaturity, remain to be investigated.

## Figures and Tables

**Table 1:** Serum and CSF biochemistry measured at day 19 in preterm pigs (Experiment 1).

**Serum**	**PRE-CON**	**PRE-HOS**	**PRE-SAL**
Albumin (g/L)	15.62 ± 2.14	15.62 ± 3.01	16.24 ± 3.32
Total protein (g/L)	26.93 ± 2.95	27.13 ± 4.82	26.96 ± 4.52
Alkaline phosphatase (U/L)	728.9 ± 298.8	974.7 ± 454.7 ^∆^	763.6 ± 284.7
Alanine aminotransferase (U/L)	28.85 ± 7.42	25.72 ± 5.80	25.78 ± 5.43
Total bilirubin (µmol/L)	2.15 ± 1.93	1.74 ± 0.99	1.98 ± 1.16
Cholesterol (mmol/L)	2.35 ± 0.46	2.42 ± 0.42	2.30 ± 0.56
Creatinine (µmol/L)	63.35 ± 34.37	59.56 ± 14.30	59.67 ± 30.64
Creatine kinase (U/L)	138.4 ± 85.0	123.9 ± 39.3	140.8 ± 76.5
Iron (µmol/L)	11.52 ± 3.96	12.74 ± 6.05	11.32 ± 5.65
Phosphate (mmol/L)	2.36 ± 0.43	2.22 ± 0.40	2.33 ± 0.38
Aspartate aminotransferase (U/L)	35.05 ± 19.09	26.67 ± 6.15 ^∆^	29.78 ± 5.43
Blood urea nitrogen (mmol/L)	8.74 ± 5.37	6.86 ± 4.30	6.23 ± 4.91
Gamma-glutamyl transferase (U/L)	26.05 ± 6.13	23.78 ± 11.24	21.94 ± 8.43
Calcium (mmol/L)	2.37 ± 0.25	2.44 ± 0.23	2.37 ± 0.24
Magnesium (mmol/L)	0.81 ± 0.13	0.86 ± 0.16	0.86 ± 0.15
Sodium (mmol/L)	140.4 ± 6.8	140.8 ± 8.0	139.0 ± 10.1
Potassium (mmol/L)	3.71 ± 0.67	3.82 ± 0.41	3.84 ± 0.47
Lactate (mmol/L)	1.56 ± 0.96	2.26 ± 2.38	2.27 ± 1.60
Glucose (mmol/L)	4.18 ± 1.58	5.32 ± 2.48 ^∆^	5.17 ± 2.77
**CSF**			
Albumin (mg/L)	12.89 ± 3.71	19.60 ± 26.11	18.57 ± 13.32
Total protein (mg/L)	177.3 ± 51.2	294.0 ± 455.1	260.0 ± 217.9
Lactate (mmol/L)	1.71 ± 0.78	1.86 ± 0.56	1.82 ± 0.66
Glucose (mmol/L)	2.28 ± 0.97	2.78 ± 0.70 ^∆^	2.74 ± 1.30

Abbreviations: CSF, cerebrospinal fluid. ^∆^ indicates a difference between PRE-CON and PRE-HOS. Values are presented as mean ± SD.

## Supplementary Materials

The following are available online at http://www.mdpi.com/2072-6643/10/10/1489/s1, Figure S1: Dual Energy X-ray absorptiometry (DEXA) (Experiment 1), Figure S2: Gut structure (Experiment 1), Table S1: Organ weights (Experiment 1), Table S2: Hematology (Experiment 1), Table S3: Organ weights (Experiment 2), Table S4: Small intestinal brush border enzyme activity and colonic microbial metabolite concentrations (Experiment 2), Table S5: Hematology (Experiment 2), Table S6: Serum and CSF biochemistry (Experiment 2).
