# Supplementary material for: Bovine Milk Oligosaccharides with Sialyllactose for Preterm Piglets
Source: Nutrients. 2018 Oct 12;10(10):1489. doi: 10.3390/nu10101489 (PMC6213258; doi:10.3390/nu10101489)
Supplement: Supplementary file 1 [file nutrients-10-01489-s001.pdf]

Supplementary Materials

Figure S1

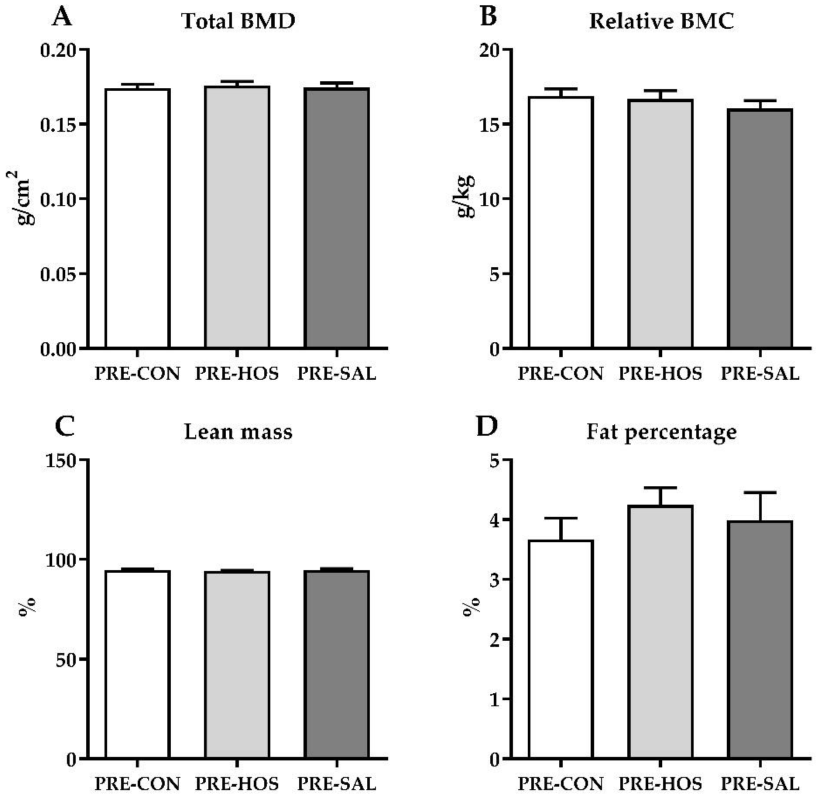

Figure S1. Dual Energy X-ray absorptiometry (DEXA) in preterm pigs (Experiment 1). (A) Bone mineral density (BMD, g/cm^2), **(B)** bone mineral content (BMC, g/kg), **(C)** lean mass percentage and **(D)** fat percentage. Values are presented as mean ± SEM.

Figure S2

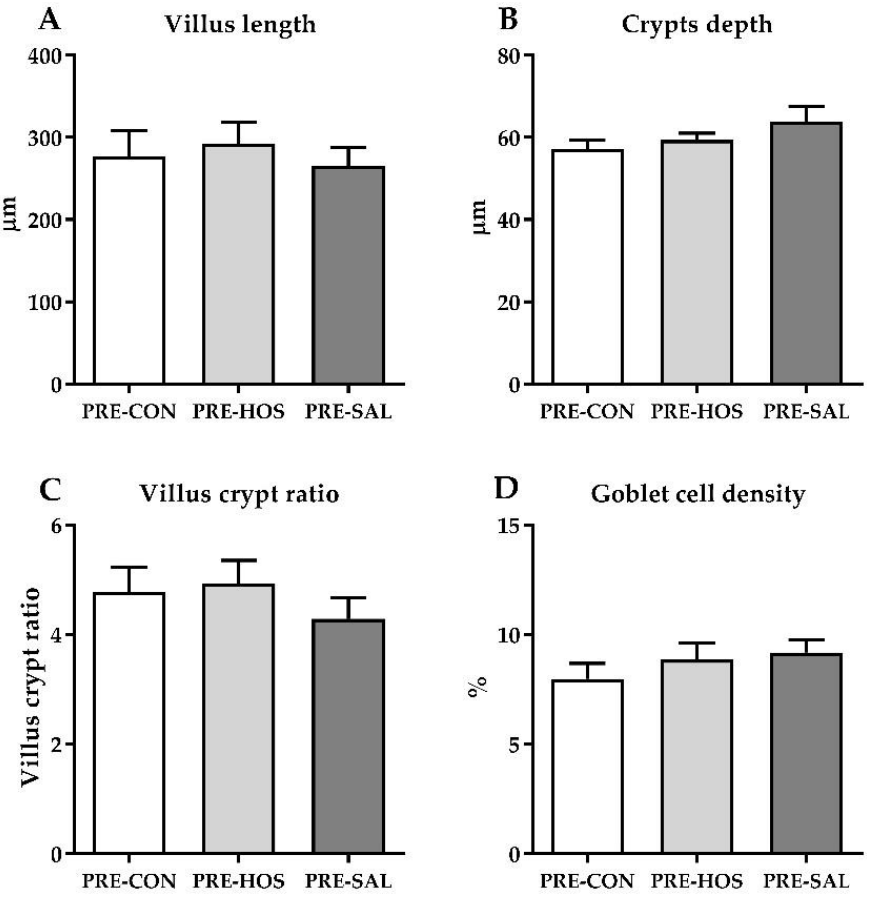

Figure S2. Gut structure in preterm pigs (Experiment 1). **(A)** Villus height, **(B)** crypt depth, and **(C)** villus/crypt ratio in the distal part of the small intestine. **(D)** Goblet cell density in colon. Values are presented as mean ± SEM.

Table S1

Organ weights at day 19 in preterm pigs (Experiment 1).

**Relative organ weights (g/kg body weight)**	**PRE-CON**	**PRE-HOS**	**PRE-SAL**
Proximal small intestine	15.0 ± 2.9	14.6 ± 2.8	14.2 ± 3.2
Middle small intestine	14.5 ± 3.3	13.9 ± 3.1	14.4 ± 3.5
Distal small intestine	16.7 ± 3.5	15.6 ± 2.6	15.4 ± 3.2
Stomach	7.6 ± 1.3	7.1 ± 1.0	7.1 ± 1.2
Colon	16.1 ± 7.7	15.6 ± 4.1	18.3 ± 10.1
Liver	22.3 ± 5.2	23.2 ± 6.5	23.1 ± 5.2
Spleen	1.9 ± 1.1	2.2 ± 1.2	2.2 ± 0.9
Heart	6.2 ± 1.0	6.3 ± 1.1	6.1 ± 1.0
Lungs	14.4 ± 2.2	16.8 ± 4.5	16.0 ± 5.0
Kidneys	7.4 ± 1.2	7.3 ± 1.3	6.9 ± 1.4
Adrenals	0.25 ± 0.06	0.22 ± 0.04	0.21 ± 0.06§
Brain	27.8 ± 10.0	27.0 ± 7.9	24.2 ± 8.0

Expressed as relative weights as g/kg body weight. § indicates difference between PRE-CON and PRE-SAL. Values are presented as mean ± SD.

Table S2

Hematology measured at day 1, 10 and 19, respectively, in preterm pigs (Experiment 1).

**Day 1**	**PRE-CON**	**PRE-HOS**	**PRE-SAL**
WBC (×10⁹/L)	2.6 ± 0.6	2.8 ± 0.4	2.7 ± 0.4
RBC (×10¹²/L)	4.2 ± 0.4	4.3 ± 0.4	4.3 ± 0.4
Hemoglobin (mmol/L)	5.5 ± 0.4	5.6 ± 0.5	5.7 ± 0.5
Hematocrit (L/L)	0.29 ± 0.02	0.29 ± 0.02	0.30 ± 0.02
Platelets (×10⁹/L)	107 ± 29	113 ± 45	132 ± 57§
Neutrophils (×10⁹/L)	0.7 ± 0.2	0.7 ± 0.3	0.7 ± 0.2
Lymphocytes (×10⁹/L)	1.8 ± 0.5	1.9 ± 0.4	1.8 ± 0.4
Monocytes (×10⁹/L)	0.08 ± 0.04	0.07 ± 0.05	0.12 ± 0.24
Eosinophils (×10⁹/L)	0.03 ± 0.02	0.02 ± 0.01	0.02 ± 0.02
Basophils (×10⁹/L)	0.02 ± 0.02	0.02 ± 0.01	0.02 ± 0.02
**Day 10**			
WBC (×10⁹/L)	5.5 ± 1.3	5.4 ± 1.6	5.6 ± 1.2
RBC (×10¹²/L)	4.4 ± 0.7	4.4 ± 0.6	4.3 ± 0.9
Hemoglobin (mmol/L)	5.1 ± 1.1	5.0 ± 0.8	5.0 ± 1.1
Hematocrit (L/L)	0.3 ± 0.1	0.3 ± 0.03	0.3 ± 0.05
Platelets (×10⁹/L)	281 ± 124	303 ± 125	331 ± 156
Neutrophils (×10⁹/L)	2.8 ± 0.8	2.7 ± 1.1	2.7 ± 0.8
Lymphocytes (×10⁹/L)	2.4 ± 0.7	2.4 ± 0.7	2.5 ± 0.6
Monocytes (×10⁹/L)	0.13 ± 0.06	0.11 ± 0.05	0.13 ± 0.08
Eosinophils (×10⁹/L)	0.10 ± 0.03	0.11 ± 0.05	0.11 ± 0.03
Basophils (×10⁹/L)	0.01 ± 0.01	0.02 ± 0.02	0.03 ± 0.03
**Day 19**			
WBC (×10⁹/L)	10.3 ± 3.5	8.7 ± 5.9	8.9 ± 5.2
RBC (×10¹²/L)	4.8 ± 0.7	4.6 ± 0.7	4.5 ± 0.7
Hemoglobin (mmol/L)	5.0 ± 0.8	4.8 ± 0.6	4.8 ± 0.7
Hematocrit (L/L)	0.26 ± 0.05	0.25 ± 0.04	0.24 ± 0.05
Platelets (×10⁹/L)	378.± 210	333 ± 184	299 ± 193
Neutrophils (×10⁹/L)	6.0 ± 2.9	4.9 ± 5.0	5.6 ± 4.3
Lymphocytes (×10⁹/L)	3.6 ± 1.1	3.3 ± 1.2	2.7 ± 1.3
Monocytes (×10⁹/L)	0.3 ± 0.2	0.2 ± 0.1	0.2 ± 0.2
Eosinophils (×10⁹/L)	0.15 ± 0.05	0.14 ± 0.14	0.11 ± 0.14
Basophils (×10⁹/L)	0.04 ± 0.05	0.03 ± 0.03	0.03 ± 0.03

Abbreviations: WBC, white blood cells; RBC, red blood cells. § indicates difference between PRE-CON and PRE-SAL. Values are presented as mean ± SD.

Table S3

Organ weights at day 19 in near-term pigs (Experiment 2).

**Relative organ weights (g/kg body weight)**	**TERM-CON**	**TERM-NAT**	**P-value**
Proximal small intestine	14.2 ± 1.7	11.7 ± 1.6	**
Middle small intestine	11.2 ± 1.2	11.8 ± 1.7	*

Distal small intestine	15.2 ± 1.4	15.7 ± 1.8	
Stomach	7.0 ± 1.0	6.1 ± 1.9	
Colon	24.5 ± 8.9	12.8 ± 3.8	**
Liver	24.9 ± 3.8	26.0 ± 2.6	
Spleen	2.9 ± 0.6	5.2 ± 1.6	***
Heart	6.5 ± 0.5	6.4 ± 0.8	
Lungs	13.4 ± 1.9	16.6 ± 4.5	*
Kidneys	8.3 ± 1.2	7.2 ± 0.5	
Adrenals	0.20 ± 0.03	0.12 ± 0.02	***
Brain	23.6 ± 4.4	11.0 ± 4.9	***

Expressed as relative weights as g/kg body weight. Values are presented as mean ± SD.

Table S4

Small intestinal brush border enzyme activity and colonic microbial metabolite concentrations in near-term pigs (Experiment 2)

**Brush border enzymes[1] (U/g)**	**TERM-CON**	**TERM-NAT**	**P-value**
Sucrase	0.29 ± 0.17	10.4 ± 2.46	***
Maltase	3.35 ± 1.30	27.0 ± 8.04	***
Lactase	2.72 ± 2.12	53.2 ± 9.83	***
Aminopeptidase N	2.43 ± 0.56	8.44 ± 2.24	***
Aminopeptidase A	1.31 ± 0.51	5.61 ± 0.93	***
Dipeptidyl peptidase IV	0.89 ± 0.24	3.77 ± 0.68	***
**Microbial metabolites (mmol/L)**			
Acetic acid	65.9 ± 34.9	183.1 ± 103.5	**
Propionic acid	0.68 ± 0.74	26.3 ± 16.3	***
Butyric acid	1.87 ± 1.45	9.39 ± 7.01	***
Valeric acid	0.09 ± 0.20	4.34 ± 2.50	***
Propanediol	1.00 ± 0.93	2.15 ± 2.55	
Methyl Butanoic Acid	0.07 ± 0.04	1.56 ± 0.77	***
Lactate	41.5 ± 38.9	4.37 ± 12.3	**
Σ (SCFA and lactate)	111 ± 589	231 ± 131	

[1] Day 19 brush border enzyme activity in the middle part of the small intestine. Values are presented as mean ± SD.

Table S5

Hematology measured at day 1, 10 and 19, respectively in near-term pigs (Experiment 2)

	Day 1	Day 10		P-value	Day 19		P-value
	**TERM-CON**	**TERM-CON**	**TERM-NAT**		**TERM-CON**	**TERM-NAT**	
WBC (×10^9/L)	4.0 ± 1.0	6.6 ± 1.9	15.0 ± 6.7	**	6.6 ± 3.0	8.8 ± 6.0	
RBC (×10^{12}/L)	5.2 ± 0.5	5.8 ± 0.7	4.9 ± 0.6	**	5.3 ± 0.7	4.7 ± 0.7	*
Hemoglobin (mmol/L)	6.3 ± 0.6	6.4 ± 0.6	5.8 ± 0.6	**	5.7 ± 0.7	4.5 ± 1.1	**
Hematocrit (L/L)	0.34 ± 0.03	0.3 ± 0.03	0.3 ± 0.03		0.30 ± 0.04	0.24 ± 0.05	**
Platelets (×10^9/L)	205 ± 45	208 ± 80	539 ± 151	***	201 ± 128	527 ± 297	**
Neutrophils (×10^9/L)	2.0 ± 0.8	3.0 ± 1.4	8.4 ± 6.6	*	2.7 ± 1.9	4.4 ± 3.6	
Lymphocytes (×10^9/L)	1.9 ± 0.5	3.2 ± 1.4	5.8 ± 2.0	**	3.4 ± 1.2	3.8 ± 4.3	
Monocytes (×10^9/L)	0.07 ± 0.06	0.19 ± 0.09	0.42 ± 0.15	***	0.3 ± 0.2	0.2 ± 0.3	
Eosinophils (×10^9/L)	0.03 ± 0.04	0.04 ± 0.05	0.04 ± 0.03		0.05 ±0.05	0.06 ± 0.05	
Basophils (×10^9/L)	0.01 ± 0.01	0.02 ± 0.01	0.10 ±0.06	***	0.04 ± 0.02	0.11 ± 0.29	

Abbreviations: WBC, white blood cells; RBC, red blood cells. Values are presented as mean ± SD.

Table S6

Serum and CSF biochemistry measured at day 19 in near-term pigs (Experiment 2)

**Serum**	**TERM-CON**	**TERM-NAT**	**P-value**
Albumin (g/L)	15.39 ± 1.74	27.47 ± 6.27	***
Total protein (g/L)	25.14 ± 1.87	42.31 ± 5.80	***
Alkaline phosphatase (U/L)	878 ± 334	3335 ± 1427	***
Alanine aminotransferase (U/L)	30.86 ± 5.17	31.42 ± 9.13	
Total bilirubin (µmol/L)	2.56 ± 0.98	3.09 ± 1.09	
Cholesterol (mmol/L)	1.75 ± 0.32	3.21 ± 0.52	***
Creatinine (µmol/L)	47.64 ± 7.37	76.92 ± 11.41	***
Creatine kinase (U/L)	222 ± 110	584 ± 226	***
Iron (µmol/L)	9.09 ± 3.28	5.87 ± 7.04	*
Phosphate (mmol/L)	2.50 ± 0.34	3.12 ± 0.67	*
Aspartate aminotransferase (U/L)	28.50 ± 5.49	37.25 ± 9.37	*
Blood urea nitrogen (mmol/L)	4.25 ± 3.09	3.40 ± 0.80	
Gamma-glutamyl transferase (U/L)	18.00 ± 4.84	26.58 ± 7.72	**
Calcium (mmol/L)	2.41 ± 0.13	2.91 ± 0.18	***
Magnesium (mmol/L)	0.80 ± 0.05	1.07 ± 0.17	***
Sodium (mmol/L)	139.5 ± 2.3	148.0 ± 5.9	***
Potassium (mmol/L)	4.03 ± 0.44	4.10 ±0.67	
Lactate (mmol/L)	1.49 ± 0.45	3.54 ± 5.10	
Glucose (mmol/L)	6.25 ± 2.01	8.74 ± 3.92	
**CSF**			
Albumin (mg/L)	8.99 ± 3.01	26.08 ± 10.06	***
Total protein (mg/L)	144.7 ± 33.0	222.0 ± 60.4	**
Lactate (mmol/L)	1.90 ± 0.64	2.32 ± 1.46	

Glucose (mmol/L)	3.32 ± 0.96	3.75 ± 0.86

Abbreviations: CSF, cerebrospinal fluid. Values are presented as mean ± SD.
